# Concerted action of berberine in the porcine intestinal epithelial model IPEC‐J2: Effects on tight junctions and apoptosis

**DOI:** 10.14814/phy2.15237

**Published:** 2022-04-05

**Authors:** Valeria Cornelius, Linda Droessler, Elisa Boehm, Salah Amasheh

**Affiliations:** ^1^ Department of Veterinary Medicine Institute of Veterinary Physiology Freie Universität Berlin Germany

**Keywords:** apoptosis, barrier function, berberine, claudins, IPEC‐J2, tight junction

## Abstract

The plant alkaloid berberine has been shown to have many beneficial effects on human health. This has led to its use as a treatment for various cancer types, obesity, and diabetes. Moreover, a described barrier‐strengthening effect in human cancer cell lines indicates that it might be useful for the treatment of inflammatory bowel disease. Detailed information regarding its effects on intestinal epithelium remains limited. In our current study, we describe the impact of berberine on a non‐transformed porcine small intestinal epithelial cell model, IPEC‐J2. Incubation of IPEC‐J2 monolayers with berberine revealed dose‐ and time‐dependent effects on barrier properties. A viability assay confirmed the specific effect of berberine on the apoptotic pathway, paralleled by the internalization of the sealing tight‐junction (TJ) proteins claudin‐1, claudin‐3, and occludin within 6 h. Hence, the barrier function of the cells was reduced, as shown by the reduced transepithelial electrical resistance and the increased [^3^H]‐D‐Mannitol flux. A decrease of claudin‐1, claudin‐3, and occludin expression was also observed after 24 h, whereas ZO‐1 expression was not significantly changed. These data indicate an early effect on both cell viability and barrier integrity, followed by a general effect on TJ architecture. The intracellular co‐localization of claudin‐1 and occludin or claudin‐3 and occludin points to an initial induction of apoptosis accompanied by the internalization of sealing TJ proteins. Although barrier strengthening has been reported in cancerogenic epithelial models, our results show a barrier‐weakening action, which represents a new aspect of the effect of berberine on epithelia. These results agree with the known toxic potential of plant alkaloids in general and show that berberine is also capable of exerting adverse effects in the intestinal epithelium.

## Graphical abstract

The plant alkaloid berberine has been shown to have many beneficial effects on human health, which led to the use as a treatment against different cancer types, obesity, and diabetes. Our current study describes the impact of berberine in a non‐transformed porcine small intestinal epithelial cell model, IPEC‐J2. Our results are in accordance with the known toxic potential of plant alkaloids in general and show that berberine also exerts the potential of adverse effects in the intestinal epithelium.

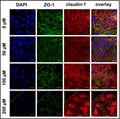

## 1 INTRODUCTION

Berberine occurs naturally in the roots of various *Berberis* species (Habtemariam, 2016) and has been used as a treatment against gastrointestinal disorders for centuries in traditional Chinese medicine (Chen et al., 2014). The plant alkaloid has been the focus of scientific research during the few last years, and its many beneficial effects on human health were identified (Jin et al., 2016; Sudomova et al., 2021; Yang et al., 2021). These benefits include the potential of berberine as a treatment against various cancer types, obesity, Alzheimer´s disease, atherosclerosis, and diabetes.

In the intestine, different effects of berberine have been described. It reduces gastrointestinal motility by inhibiting the myosin light chain kinase (Cao et al., 2013; Chen et al., 2013; Xu et al., 2017) and by decreasing the pacemaker potential within the interstitial cells of Cajal (Kim et al., 2016). Furthermore, it has anti‐secretory (Tai et al., 1981; Taylor & Baird, 1995; Zhu & Ahrens, 1983), anti‐inflammatory, and anti‐microbial properties (Chen et al., 2014).

Moreover, the effect of berberine on the paracellular intestinal barrier function has been analyzed in a variety of intestinal models (Radloff et al., 2019).

A barrier‐strengthening effect of berberine has been demonstrated in experiments on cultures of human intestinal epithelial cells, namely Caco‐2 and HT29/B6 cells (Amasheh et al., 2010; Gu et al., 2009; Valenzano et al., 2015). Furthermore, various barrier‐disturbing effects of factors such as uremia (Yu et al., 2016) (rat model), dextran sulfate sodium (Zhang et al., 2017) (mouse model), peritoneal air exposure (Tan et al., 2015) (rat model), LPS (Gu et al., 2011) (mouse model) or TNFα (Cao et al., 2013) (CaCo‐2 cells) (Amasheh et al., 2010), (HT29/B6 cells and rat model) was mitigated by berberine. Therefore, berberine is a promising candidate as a treatment against intestinal diseases that are accompanied by inflammation e.g. inflammatory bowel diseases (Habtemariam, 2016).

The paracellular barrier properties of epithelia are determined by the expression of tight junction (TJ) proteins, mainly those of the claudin protein family (Markov et al., 2015). The claudin subset differs between the different sections of the intestine (Markov et al., 2010). Most claudins have a sealing function, for example, claudin‐1, −3, −4, −5, and −7, which can all be found in the small and the large intestine. Claudin‐2 is a pore‐forming claudin and is expressed in the small but not in the large intestine (Gunzel & Yu, 2013; Markov et al., 2010). Other essential TJ proteins are occludin, which has a sealing function and can be involved in signaling processes, and zonula‐occludens‐protein 1 (ZO‐1), an intracellular scaffolding protein that connects the claudins with the cytoskeleton of the cells (Balda & Matter, 2008). Cell culture experiments with Caco‐2 and HT29/B6 cells indicate that berberine is able to modify the expression pattern of claudins. In both cell lines, claudin‐2 has been shown to be reduced, while claudin‐1 was upregulated in HT29/B6 cells, and claudin‐3 and −5 were increased in Caco‐2 cells (Amasheh et al., 2010; Gu et al., 2009; Valenzano et al., 2015).

Our aim, in this study, was to analyze the effect of berberine on the barrier function of the porcine small intestinal cell line IPEC‐J2. These cells were isolated from the jejunum of a neonatal piglet and have been previously characterized in detail by Nossol et al. (2015); Schierack et al. (2006); Vergauwen et al. (2015); Zakrzewski et al. (2013). As the cells are non‐transformed and non‐cancerogenic and grow as a polarized monolayer with typical properties of the small intestinal epithelium, their physiology and signaling reflects that of native epithelium (Droessler et al., 2021; Gonzalez et al., 2015; Schierack et al., 2006; Vergauwen et al., 2015). Moreover, as the human and porcine intestine have many anatomical and physiological similarities, and as both species are omnivorous, the porcine intestine and the IPEC‐J2 cell line represent a useful model for the human intestine in health and disease and with regard to susceptibility to affecting agents (Nossol et al., 2015; Zakrzewski et al., 2013). In this study, we aimed to analyze the effect of berberine on the barrier function of the porcine small intestinal, *non*‐*transformed* cell line IPEC‐J2 and our hypothesis was that this could be characterized in detail reflecting intestinal epithelial physiology.

## 2 MATERIAL & METHODS

### 2.1 Cell culture and experiments

The non‐transformed cell line IPEC‐J2 was obtained from DSMZ and cultured in Dulbecco's MEM/Ham's F‐12 (Biochrom) supplemented with 10% porcine serum (Sigma Aldrich) and 1% penicillin‐streptomycin (Sigma Aldrich) at 37°C in a humidified 5% CO_2_ atmosphere. Routinely, the medium was changed every 2–3 days, and the cells were split once a week. For incubation experiments, cells were used between passages 7 and 15. 10^5^ cells were seed on semipermeable PCF‐culture plate inserts that had a size of 12 mm and a pore diameter of 0.45 µM (Millipore) and that were placed in 12‐well cell culture plates. After 14 day of growth, the transepithelial electrical resistance (TEER) of the cell monolayers was measured with a chopstick electrode and an epithelial volt‐ohm meter (EVOM) (World Precision Instruments). Once the TEER values were stable, we initiated incubation of the cells with berberine. Stock solutions of berberine chloride (Sigma Aldrich), dissolved in DMSO (Sigma Aldrich), were prepared and added to complete cell culture medium, resulting in solutions with 50, 100, or 200 µM berberine and for each of them 0.2% DMSO; controls were treated with 0.2% DMSO. These solutions were added to the apical and the basolateral compartment to give stable conditions during the time course of the experiment, in accordance with previous studies (Amasheh et al., 2010). TEER was measured directly before the addition of berberine and after 4, 6, and 24 h. For each condition, three filters were used, and the whole setup was repeated 5 times, although not all inserts were measured at every time point. After 6 h or 24 h, the cells were lysed for protein analysis or fixed for immunostaining.

### 2.2 Paracellular permeability measurements

We used [^3^H]‐d‐Mannitol (PerkinElmer) to measure the unidirectional paracellular tracer flux from the apical to basolateral compartments of the cells during incubation with berberine for 6 h. The cells were seeded onto semipermeable cell culture inserts and incubated with 50 μM, 100 μM, or 200 μM berberine as described above. [^3^H]‐D‐Mannitol (0.18 μCi) was added to the apical compartment of the cell culture filters, and samples of 50 μl were taken directly and after 6 h. Specific tracer activity was calculated using Equation (1). Every 2 h, samples of 300 μl were removed from the basolateral part of the cell culture plates, and fresh media containing the respective concentration of berberine was added immediately to guarantee unchanged incubation conditions. An Aquasafe 300plus liquid scintillation cocktail (Perkin Elmer) was added to the samples after removal. Samples were measured by a TriCarb 4910TR liquid scintillation counter (PerkinElmer). Finally, the paracellular unidirectional tracer flux was calculated using Equation (2) shown below.

(1) $$\text{spec}.\;\text{activity}\left[\text{nmol}\right]=\frac{\text{mean}\;({\text{counts}}_{\text{donor}\;\text{side}})}{{\text{concentration}}_{\text{donor}\;\text{side}}\times {\text{volume}}_{\text{donor}\;\text{side}}}$$

(2) $$\begin{array}{c} J\left[\text{nmol}\times {\text{cm}}^{‐2}\times h^{‐1}\right]\\ =\frac{{\text{counts}}_{t}\times \frac{V_{\text{chamber}}}{V_{\text{sample}}}‐{\text{counts}}_{t‐1}\times \frac{V_{\text{chamber}}‐V_{\text{dilution}}}{V_{\text{sample}}}}{\text{specific}\;\text{activity}\times \text{area}\times \text{time}} \end{array}$$

### 2.3 Viability assay

An ApoToxGlo^TM^ Triplex assay (Promega GmbH) was performed as instructed by the manufacturer in order to analyze the cell viability, the toxicity, and the activation of caspase‐3 and −7 as indicators for the induction of apoptosis in the same well. The cells were cultivated in a 96‐well plate with 10^5^ cells per well for 3–5 days, and the assay was performed after 6 h of incubation with the control medium containing 0.2% DMSO and berberine concentrations from 25 to 200 µM. Two independent assays with duplets were performed, and the data are presented as percentages of the control value.

### 2.4 Protein extraction and quantification

After 6 h or 24 h of incubation, the cells were rinsed in PBS, ice‐cold lysis buffer was added, and the cells were detached mechanically. The lysis buffer contained in mmol·L^−1^: HEPES (25), NaF (25), EDTA (2), 1% SDS, and enzymatic protease inhibitors (Complete EDTA‐free, Roche). Following a 30‐min incubation on ice, the samples were homogenized. For protein quantification, Biorad DC‐dye (Bio‐Rad Laboratories GmbH) was used as instructed, and proteins were detected by an EnSpire Multimode Plate Reader (PerkinElmer).

### 2.5 Immunoblotting and densitometry

Twenty micrograms of protein and Laemmli buffer (Bio‐Rad Laboratories GmbH) were mixed and loaded onto a 10% TGX Stain‐Free FastCast gel (Bio‐Rad Laboratories GmbH). After electrophoresis at 150 V for 60 min, the proteins were transferred to a PVDF membrane (Bio‐Rad Laboratories GmbH, Munich, Germany) for 90 min at 100 V. The membrane was blocked for 60 min in 5% milk (in Tris‐buffered saline [TBS] with 0.1% Tween 20 [TBS‐T]) and incubated with the primary antibodies raised against claudin‐1, claudin‐3, occludin, or ZO‐1 (Thermo Fisher) overnight at 4°C. After three washing steps with TBS‐T, the blots were incubated with horseradish‐peroxidase‐conjugated secondary antibodies raised against mouse or rabbit (Cell Signaling Technology) for 1 h at room temperature. Following the detection of the total protein amount with the ChemiDoc MP Luminescence imager, Clarity Western ECL Blotting Substrate (Bio‐Rad Laboratories GmbH) was used to visualize the protein bands. Blots were then rinsed in TBS, blocked again, and incubated with another primary antibody (rabbit, if mouse was used in the first step, and vice versa). Because of the NaN_3_ in the primary antibody solution, the horseradish‐peroxidase from the secondary antibody was destroyed during the incubation, and thus, the signal from the first incubation step was no longer detectable. The subsequent steps were as described above. The potential loss of protein during washing steps was acknowledged by a new determination of the total protein amount before the visualization of the protein bands. For densitometry, the signals were normalized to the total protein amount detected directly before the visualization and are expressed relative to the control values, which were set to 100%.

### 2.6 Immunocytochemistry

Following 6 h or 24 h of incubation, the cell culture medium was removed, and the cells were fixed with ice‐cold methanol for 10 min at −20°C. After being rinsed, the cells were blocked with 5% goat serum (Pan Biotech) in PBS for 60 min. The membranes were cut out of the cell culture inserts, cut into two halves, and placed inside the primary antibody solutions overnight at 4°C. Primary antibodies raised against claudin‐1, claudin‐3, occludin, and ZO‐1 were used (1:50 ‐ Thermo Fisher). Following a washing step in blocking solution, samples were incubated with goat anti‐mouse Alexa Fluor‐488, goat anti‐rabbit Alexa Fluor‐594 (1:1000, Thermo Fisher), and DAPI (1:5000) for 60 min at 37°C. After being rinsed with blocking solution, the membranes were mounted with ProTaqs Mount Fluor (Biocyc), analyzed, and visualized using a Zeiss 710 confocal laser scanning microscope (Zeiss). The settings of the microscope were identical for each staining series.

### 2.7 TUNEL assay

To visualize apoptotic cell nuclei, a Click‐iT^TM^ TUNEL assay with Alexa Flour 647 (Thermo Fisher) was performed. IPEC‐J2 cells were seeded on coverslips and cultured for 14 days. Following 24 h of incubation with berberine, the cells were fixed with 4% paraformaldehyde (Roti‐Histofix). After the TUNEL assay had been performed as instructed, DAPI and ZO‐1 were stained as described above.

### 2.8 Statistical analysis

Results from TEER measurements, viability assays, and densitometry are expressed as the mean ±standard error of the mean, with *n* being the number of cell culture inserts, if not indicated differently. When indicated, relative values were used for statistical evaluation.

Statistical testing was performed with one‐way ANOVA and Dunnett´s test for multiple comparisons; the statistically significant difference between the groups was determined with one‐way ANOVA for each time point and Dunnett´s test was used for comparisons between the control and the treated groups.

For the paracellular permeability measurement, data are reported as the median and interquartile range (IQR), and Kruskal‐Wallis tests were performed followed by Dunn´s multiple comparison test against the controls.

Values below *p* = 0.05 were considered to be statistically significant. MS Excel 2016 and JMP Pro 15 were used for the analysis. Figures were created with MS Office or JMP Pro 15.

## 3 RESULTS

### 3.1 TEER measurement

**FIGURE 1:**
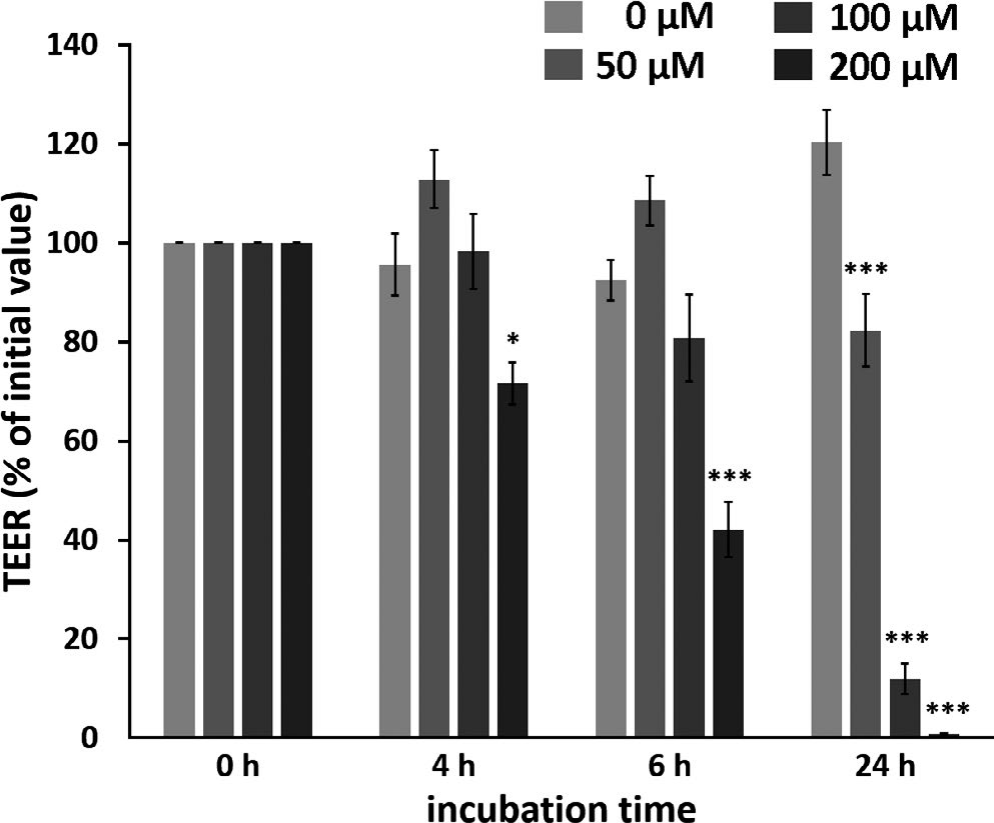
Dose‐ and time‐dependent effects of berberine on IPEC‐J2 monolayers. Berberine was used at concentrations of 50, 100, and 200 µM, and all solutions contained 0.2% DMSO. TEER was measured before the addition of berberine (*n* = 21–24), and this value was set to 100%. Every subsequent value was calculated as a percentage of this initial value. After 4 h (*n* = 16–19), 6 h (*n* = 15), and 24 h (*n* = 13–16), the TEER value was measured and compared with that of the respective controls. Values are given as mean ± SEM, and asterisks indicate significant difference from control (with * = *p* <0.05 and *** = *p* <0.001)

Incubation of IPEC‐J2 monolayers with the various concentrations of berberine induced a dose‐dependent decrease of TEER (Figure 1). After 4 h of incubation, 200 µM Berberine led to a decrease of TEER to 71.7 ± 4.3% of initial value; this dropped to 42.0 ± 5.6% after 6 h (*p* = 0.0166, *n* = 16–19 and *p* < 0.0001, *n* = 15, respectively). By 24 h of incubation, values were lower for all tested concentrations of berberine compared with the control (0 µM: 120.4 ± 6.6%; 50 µM: 82.4 ± 7.4%, *p* < 0.0001; 100 µM: 11.9 ± 3.1%, *p* < 0.0001; 200 µM: 0.8 ± 0.3%, *p* < 0.0001; *n* = 13–16, detailed results are given in Table S1).

### 3.2 Paracellular permeability measurements

**FIGURE 2:**
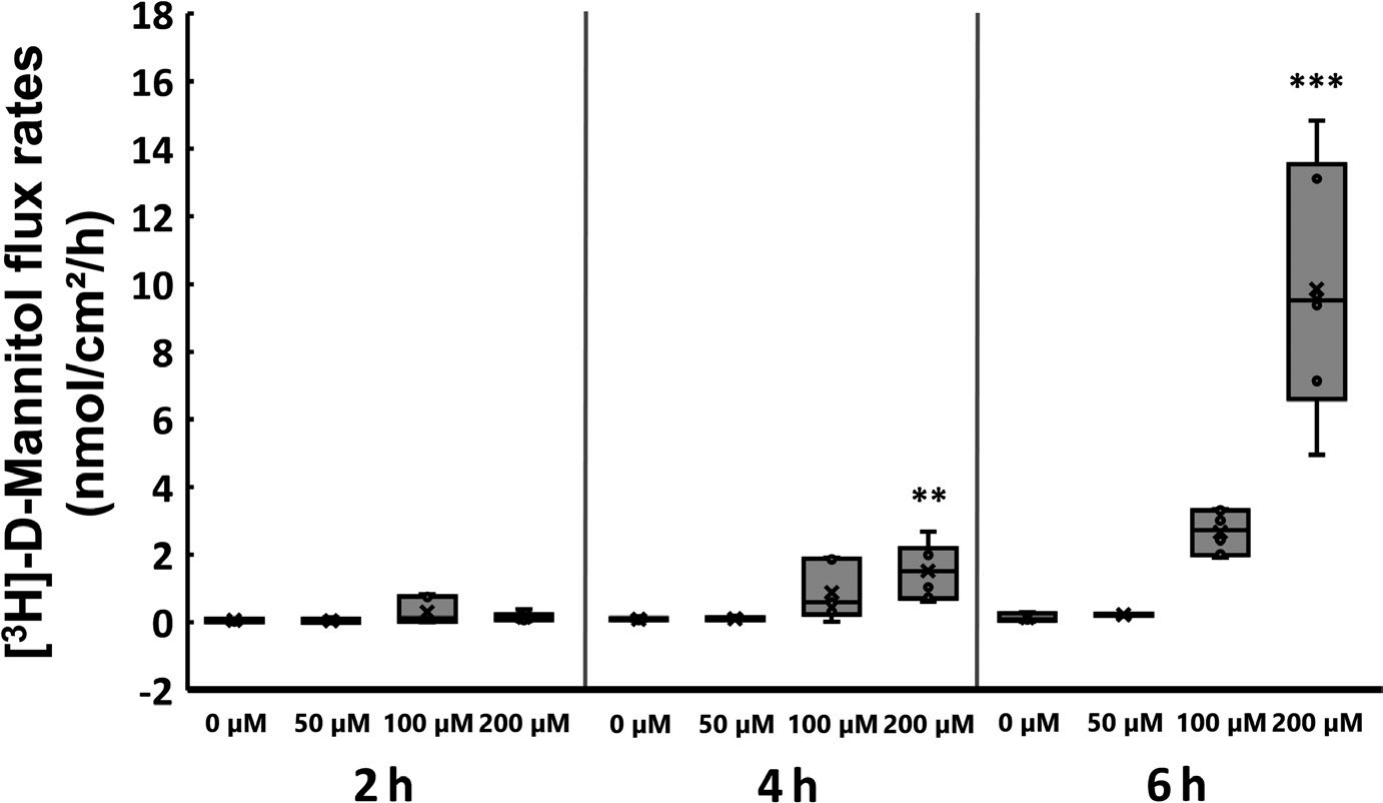
Effects of berberine on [^3^H]‐D‐Mannitol flux (nmol/cm²/h). The [^3^H]‐D‐Mannitol flux increased after 4 h incubation with 200 µM berberine and was nearly 10 times higher than that in the controls after 6 h. Boxplots show the interquartile range as a box with the median as a line inside, the mean as an X, and the single data points as circles. Whiskers represent the maximum and minimum. Asterisks indicate significant difference from the control (with ** = *p *< 0.01 and *** = *p* <0.001)

Unidirectional tracer flux analysis revealed a significant increase of [^3^H]‐d‐Mannitol flux from the apical to basolateral compartment of the cell culture inserts for the 200 µM berberine‐treated samples (Figure 2). No effect of berberine on [^3^H]‐d‐Mannitol flux could be detected during the first 2 h of incubation. After 4 h, 200 µM berberine led to an increased flux of 1.54 nmol/cm²/h, IQR: 0.73–2.21 (control: 0.1 nmol/cm²/h, IQR: 0.08–0.15, *p* = 0.0084, *n* = 5–6). The flux increased even more during the next two incubation hours. We observed a tendency to flux increase with 100 µM berberine incubation for 6 h (2.72 nmol/cm²/h, IQR: 1.98–3.31, *p* = 0.07) and a highly significant effect with 200 µM berberine (9.54 nmol/cm²/h, IQR: 6.6–13.56, *p* = 0.0004). The control group had an unchanged flux of 0.09 nmol/cm²/h, IQR: 0.04–0.25. Detailed results are given in Table S2.

### 3.3 Viability assay

**FIGURE 3:**
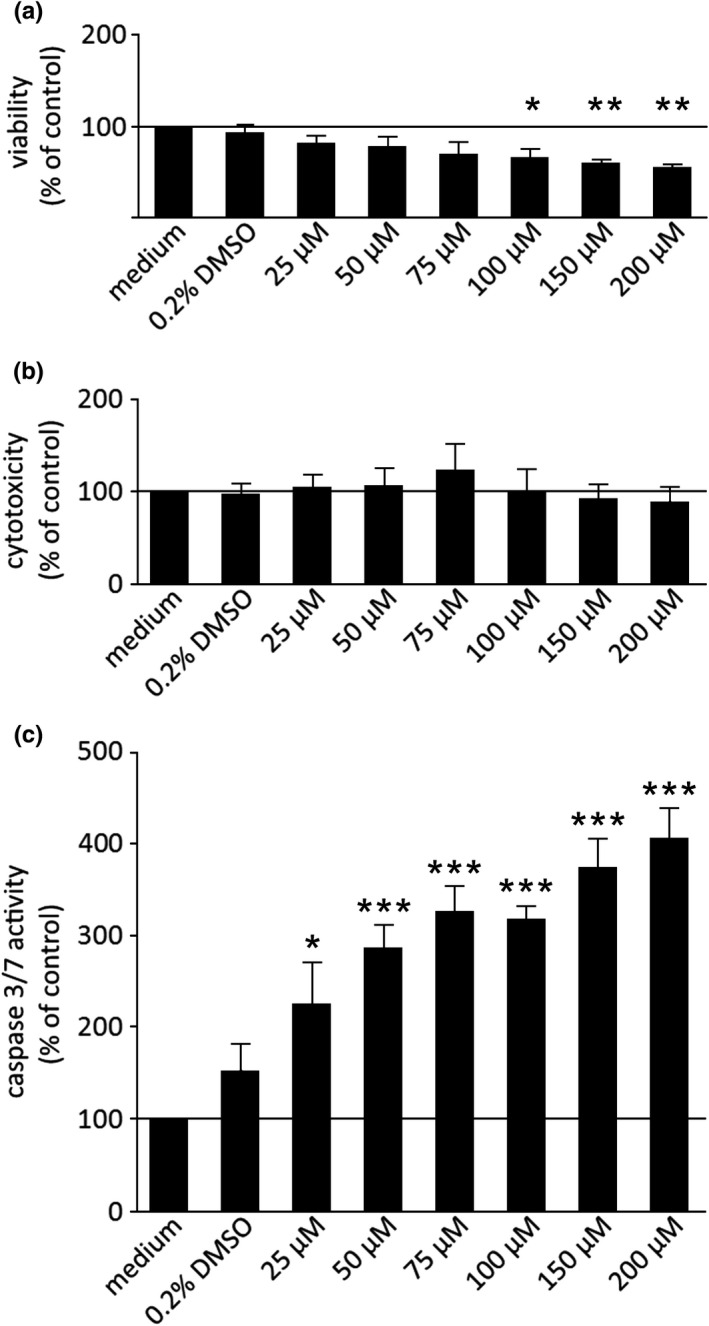
Results of the ApoToxGlo^TM^ Triplex assay showing cell viability (a), cytotoxicity (b), and caspase 3/7 activity (c) in IPEC‐J2 cells after 6 h of incubation. Berberine led to a dose‐dependent decrease of the cell viability (a) and a dose‐dependent increase of caspase‐3 and −7 activity (c). No cytotoxic effect could be observed (b). Data are expressed as % of control and are shown as mean ± SEM, *n* = 3–4. For better comparability of the percentage changes, all y‐axis were scaled identically. Values are given as mean ± SEM, and asterisks indicate significant different from control (with * = *p* < 0.05, ** = *p* < 0.01, and *** = *p* < 0.001)

Because of the drastic drop in the TEER values, an ApoToxGlo^TM^ Triplex assay was performed after 6 h of incubation time. With this assay, we were able to analyze not only the cell viability, but also the toxicity and the activation of caspase‐3 and −7 in the same well. The assay revealed a reduction of cell viability with a dose‐dependent decrease by berberine, which was significant for berberine concentrations of 100, 150, and 200 µM (100 µM: 67.5 ± 8, *p* = 0.023; 150 µM: 61.3 ± 2.5, *p* = 0.0058; 200 µM: 57.3 ± 2.5, *p* = 0.0022; Figure 3a), whereas no cytotoxic effect could be detected (Figure 3b). The dose‐dependent activation of caspase‐3 and −7 indicated the induction of apoptosis (25 µM: 225.8 ± 44.7, *p* = 0.0192; 50 µM: 286.6 ± 24.6, *p* = 0.0004; 75 µM: 326.1 ± 26.1; 100 µM: 318.1 ± 12.9; 150 µM: 373.7 ± 30.0; 200 µM: 405.6 ± 31.2; *p *< 0.0001 for 75–200 µM; Figure 3c, detailed results are given in Table S3).

### 3.4 Immunoblotting and densitometry

**FIGURE 4:**
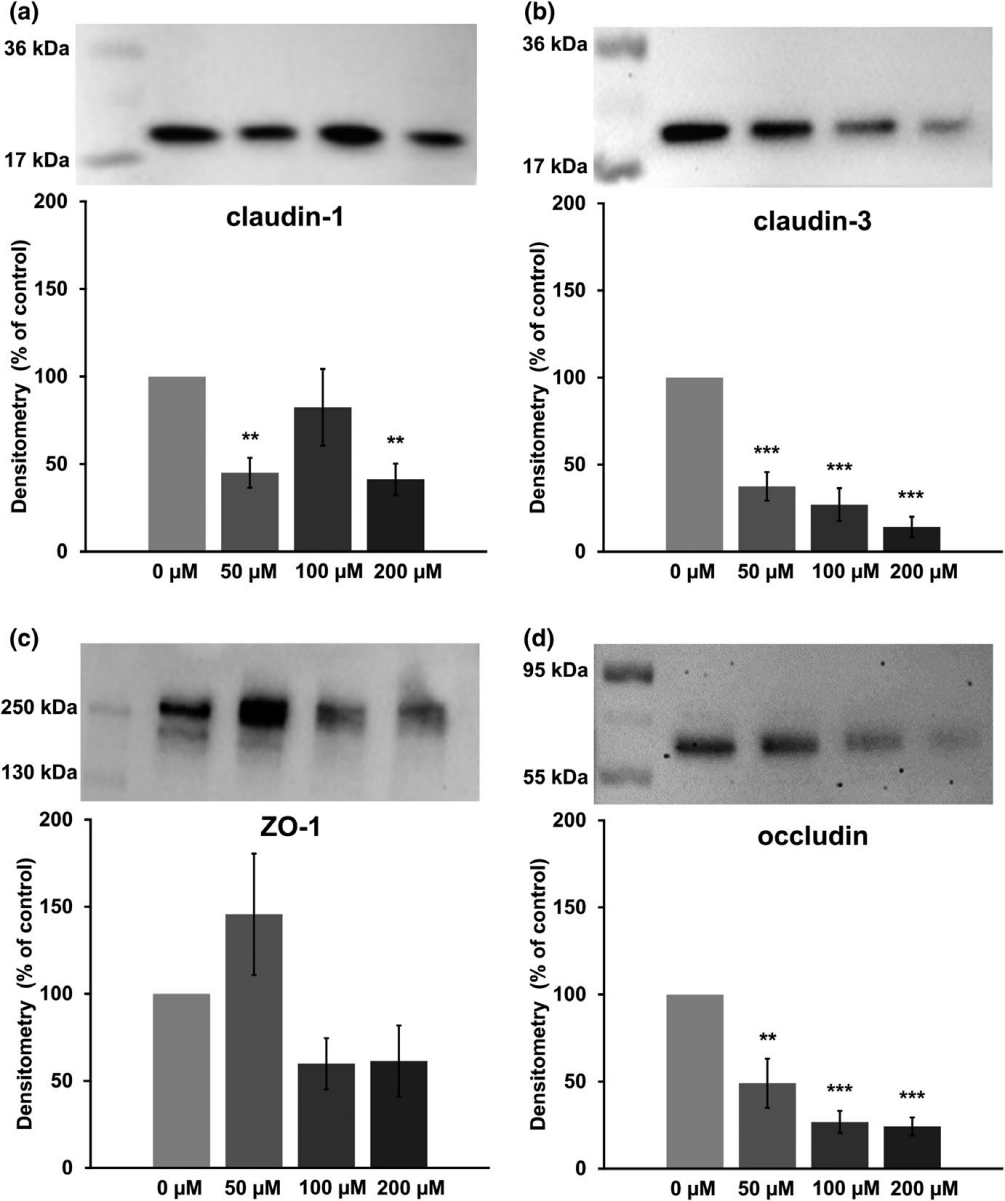
Densitometry and representative membranes with visualized protein of claudin‐1 (a, ~22 kDa), claudin‐3 (b, ~22 kDa), ZO‐1 (c, ~250 kDa), and occludin (d, ~65 kDa) after a 24‐h incubation Claudin‐1 (a) was reduced in the 50 µM and the 200 µM groups, but not in the 100 µM group. Claudin‐3 (b) and occludin (d) were reduced dose‐dependently, and ZO‐1 (c) was not changed significantly. Data are expressed as % of control, are normalized to total protein amount, and are shown as mean ± SEM, *n* = 4–6. Asterisks indicate significant difference from control with ** = *p* < 0.01 and ***=*p* < 0.001

The various TJ proteins were quantified to determine whether the drop of TEER values was attributable due to altered TJ protein expression. After immunoblotting, the protein levels were Quantified by densitometry and normalized to the total protein amount. Detection of the sealing TJ proteins revealed reduced signals of claudin‐1, with 50 and 200 µM berberine (45.0 ± 8.5%, *p* = 0.007, and 41.3 ± 9.0%, *p* = 0.004; Figure 4a), whereas 100 µM showed no significant effects (82.4 ± 22.0%, *p* = 0.6, *n* = 5–6). Furthermore, all tested concentrations led to a decrease of claudin‐3 in a dose‐dependent way (50 µM: 37.4 ± 8.2%, *p *< 0.0001; 100 µM: 27.0 ± 9.5%, *p* < 0.0001; 200 µM: 14.2 ± 5.9%, *p* < 0.0001, *n* = 5; Figure 4b). ZO‐1 was not significantly influenced by berberine (50 µM: 117.2 ± 24.7%, *p* = 0.83; 100 µM: 59.9 ± 14.7%, *p* = 0.27; 200 µM: 61,4 ± 20.4%, *p* = 0.26, *n* = 5–6; Figure 4c) and occludin was reduced dose‐dependently (50 µM: 49.1 ± 14.1%, *p* = 0.0023; 100 µM: 26.8 ± 6.4%, *p* = 0.0001; 200 µM: 24.2 ± 5.2%, *p* < 0.0001, *n* = 4; Figure 4d; detailed results are given in Table S4).

### 3.5 Immunocytochemistry and TUNEL assay

**FIGURE 5:**
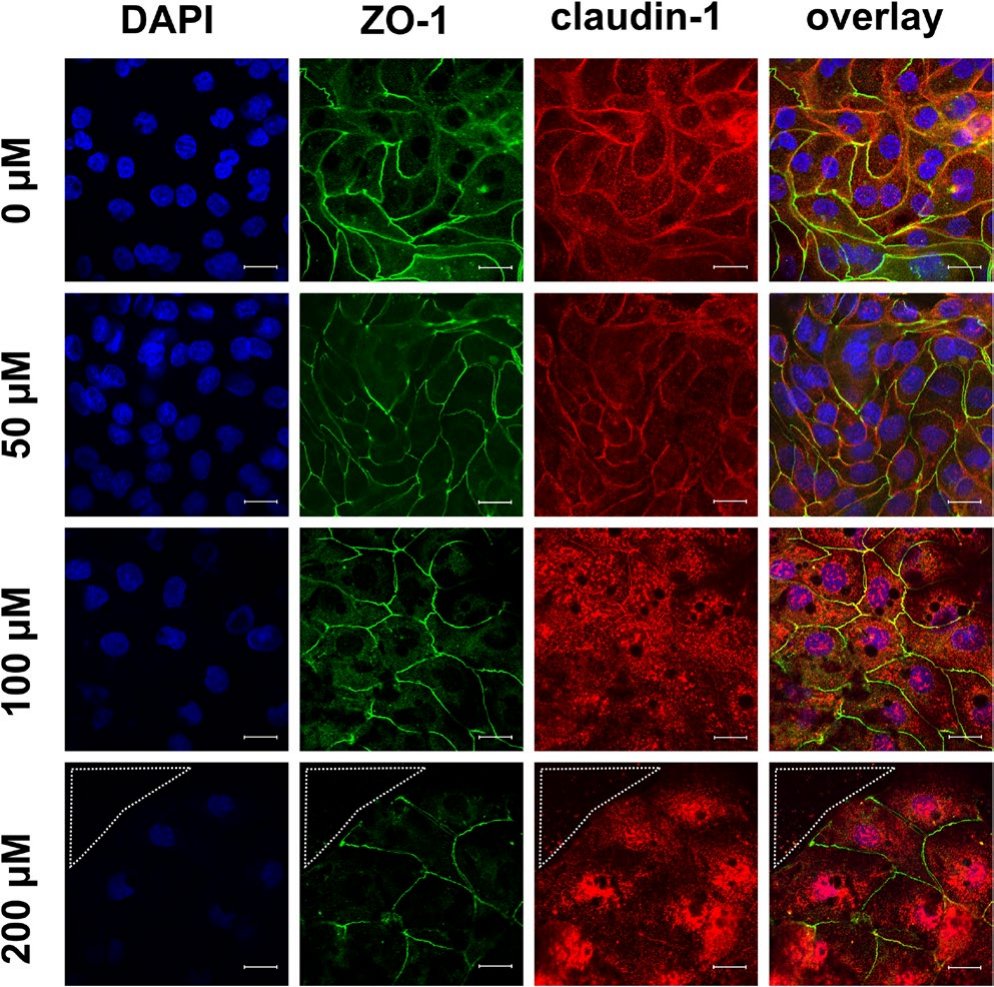
Immunocytological staining of IPEC‐J2 cells on filter membranes with antibodies raised against ZO‐1 (green) and claudin‐1 (red) after 24 h of incubation with berberine. Nuclei were stained with DAPI (blue). Under control conditions, both proteins were detected in the lateral membrane; the yellow signal in the overlay shows co‐localization. The signal for ZO‐1 was also located next to cell‐cell contacts for all concentrations, while the signal for claudin‐1 was detected more intracellularly than in the TJs of the 100 µM and the 200 µM groups. In addition, the ZO‐1 signal appears weaker with increasing berberine concentrations. The area circled by white dots shows a hole inside of the monolayer (scale bar: 20 µM, *n* = 4, representative images)

**FIGURE 6:**
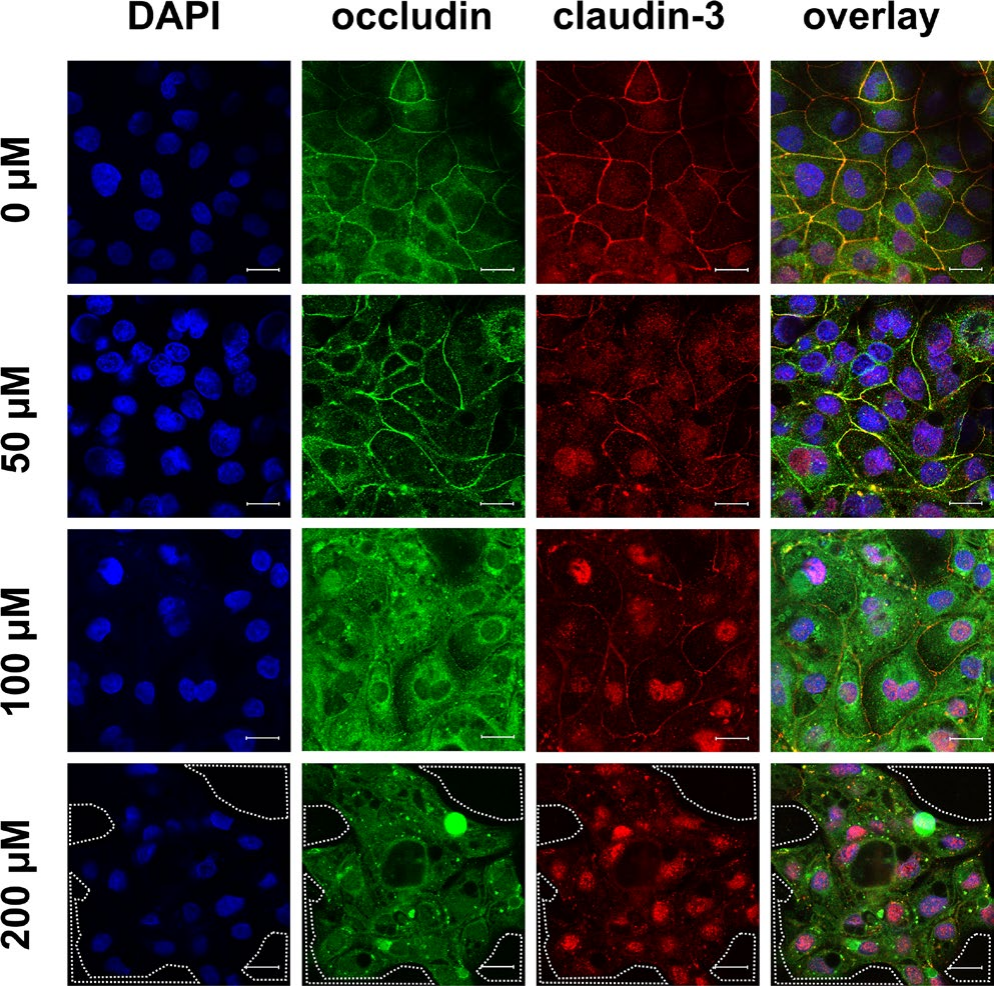
Immunocytological staining of IPEC‐J2 cells on filter membranes with antibodies raised against occludin (green) and claudin‐3 (red) after 24 h of incubation with berberine. Nuclei were stained with DAPI (blue). Under control conditions, both TJ proteins could be located in the lateral membrane; the yellow signal in the overlay showed co‐localization. The signal for occludin appeared slightly stronger with 50 µM berberine, but 100 µM lead to a more intracellular signal, which was weaker in the 200 µM group. The claudin‐3 signal became weaker dose‐dependently. The area circled by white dots shows holes inside of the monolayer (scale bar: 20 µM, *n* = 4, representative images)

**FIGURE 7:**
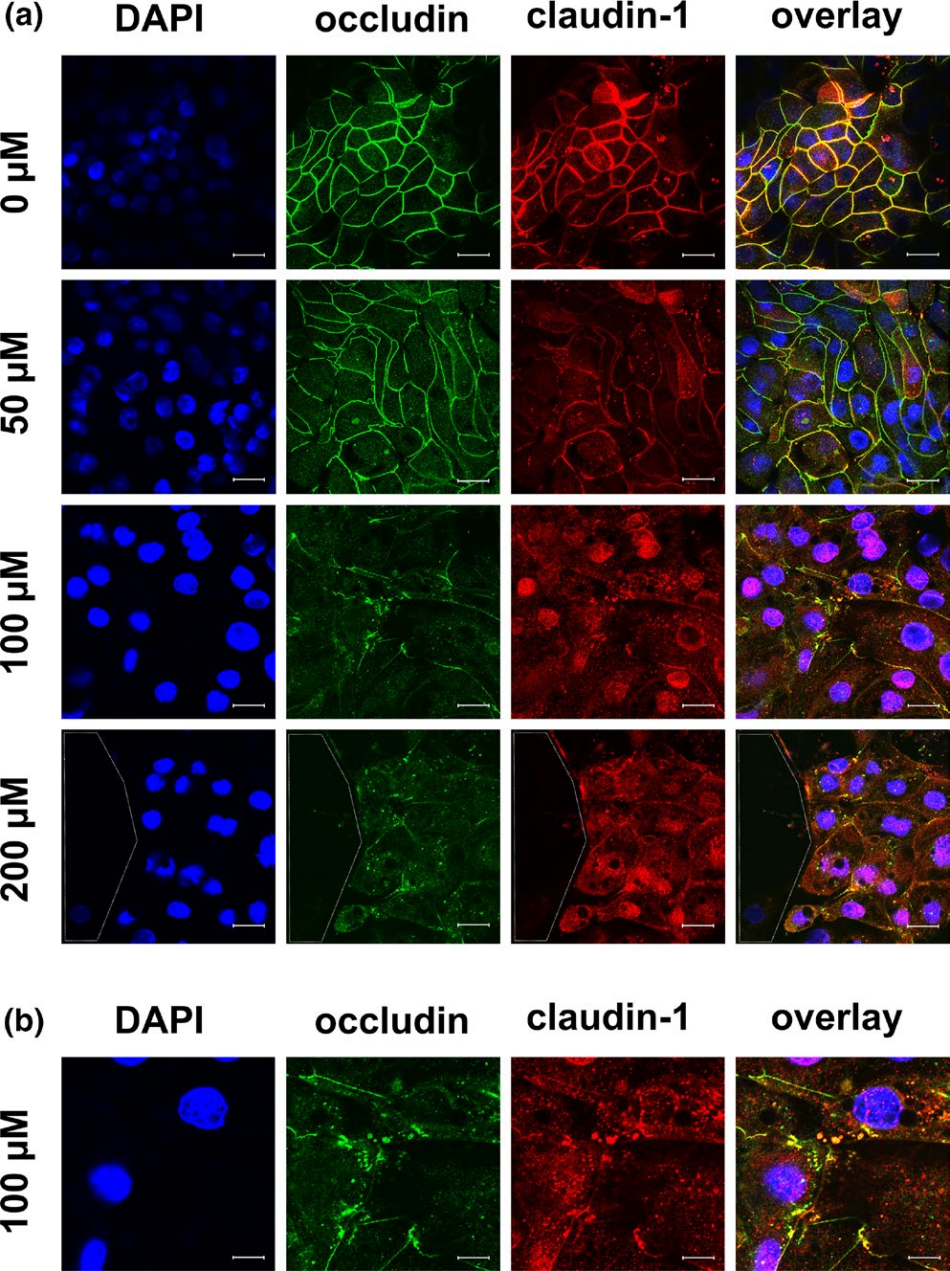
(a) Immunocytological staining of IPEC‐J2 cells on filter membranes with antibodies raised against occludin (green) and claudin‐1 (red) after 24 h of incubation with berberine. Nuclei were stained with DAPI (blue). Both TJ proteins were co‐located in the lateral membrane under control conditions, and both signals became weaker with increasing berberine concentrations. Signals for both proteins could also be found inside the cells, instead of laterally, and they were co‐localized in the sub‐membranous area. This could be seen most clearly for 100 µM and is enlarged in 7(b). The area circled by white dots shows a hole inside of the monolayer (scale bar: (a) 20 µM, (b) 40 µM, *n* = 3, representative images)

**FIGURE 8:**
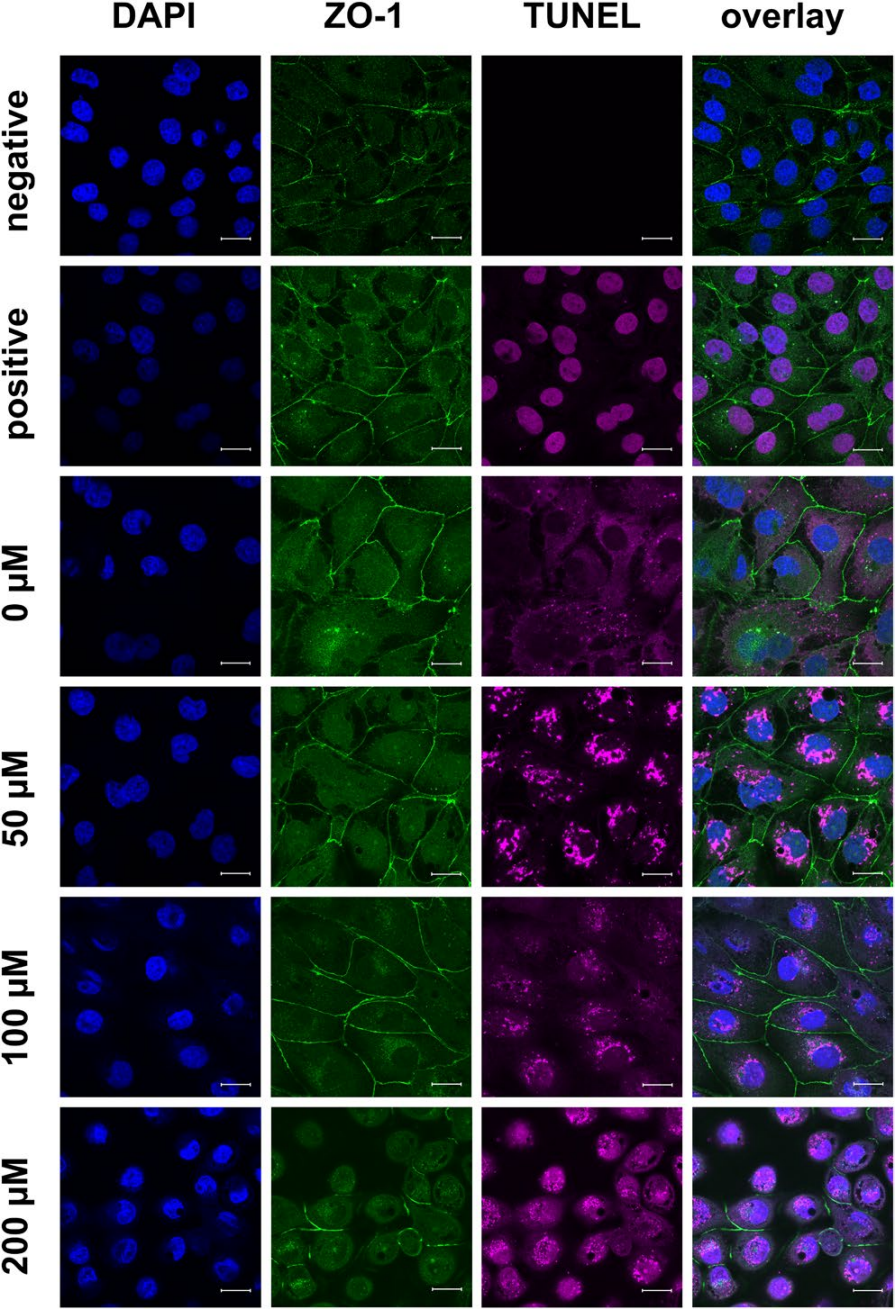
TUNEL assay (purple) and immunocytological staining of IPEC‐J2 cells on coverslips with an antibody raised against ZO‐1 (green), Nuclei were stained with DAPI (blue). The cells used as TUNEL‐positive and ‐negative controls were treated as the control (0.2% DMSO). The negative control was incubated with blocking solution during the TUNEL assay, and the positive control was treated with DNAse to induce double‐strand breakage (seen as purple staining of the nuclei). As in Figure 5, the ZO‐1 signal was located near the lateral membrane and became weaker with increasing berberine concentrations. TUNEL staining was strongest following treatment with 50 µM berberine (scale bar: 20 µM, *n* = 3, representative images)

In addition to the quantity of TJ proteins, their localization plays an important role for functionality and therefore for their physiological relevance, because a direct contribution to barrier function only can occur when the TJ proteins are integrated into the lateral membrane of the epithelial cell. Hence, we performed immunofluorescent TJ protein staining following incubation of the cells with various concentrations of berberine for 24 h. The results are shown in Figure 5 (ZO‐1 and claudin‐1), Figure 6 (occludin and claudin‐3), Figure 7 (occludin and claudin‐1), and Figure 8 (TUNEL assay and ZO‐1). The ZO‐1 signal was located near the lateral membrane, but the signal was weaker in a dose‐dependent manner. The barrier‐forming claudin‐1 was localized in the lateral membrane under control conditions, but the paracellular signal was weaker with increasing berberine concentrations, and more claudin‐1 signal was detected intracellularly. In the overlay, co‐localization was detected as a yellow signal (Figure 5). Moreover, occludin and claudin‐3 were detected in the lateral membrane and were co‐localized (Figure 6). The signal for occludin appeared to be stronger in the group of cells treated with 50 µM berberine but was internalized at higher concentrations. In the 200 µM group, the occludin signal was weaker. Similar to ZO‐1, the signal for claudin‐3, which is also a barrier‐forming claudin, decreased dose‐dependently but could not be detected in the lateral membrane of the 200 µM group.

Because of the internalization of claudin‐1 and occludin, additional staining with claudin‐1 in red and occludin in green was performed to investigate whether both TJ proteins were co‐localized intracellularly (Figure 7). Again, localization of the two TJ proteins in the lateral membrane and co‐localization under control conditions were seen. With increasing berberine concentrations, the signal became weaker and was located more intracellularly. An intracellular, sub‐membranous co‐localization of the two proteins was observed most clearly for 100 µM (the area was magnified; Figure 7b).

Moreover, a loss of cells inside the monolayer could be observed in the 200 µM group. A dotted line in the figures indicate holes in the monolayer (Figures 5, 6, 7).

With the TUNEL assay, double‐strand breaks of the DNA were stained purple (Figure 8). TUNEL‐negative control cells were untreated and incubated with blocking solution instead of the TUNEL assay reagents, and TUNEL‐positive control cells were also left untreated. The double‐strand breakage was induced with DNAse after fixation. The strongest signals could be found with 50 µM berberine, whereas the signal with 100 µM was rather weak. Treatment with 200 µM berberine gave more intense staining, but this was still weaker than that for 50 µM. Quantification of the fluorescent signals was not performed because of the background signal for cells, which were stained on the membrane of the cell culture insert (Figures 5, 6, 7), and because of the autofluorescence attributable to the formalin fixation used in the TUNEL assay (Figure 8).

**FIGURE 9:**
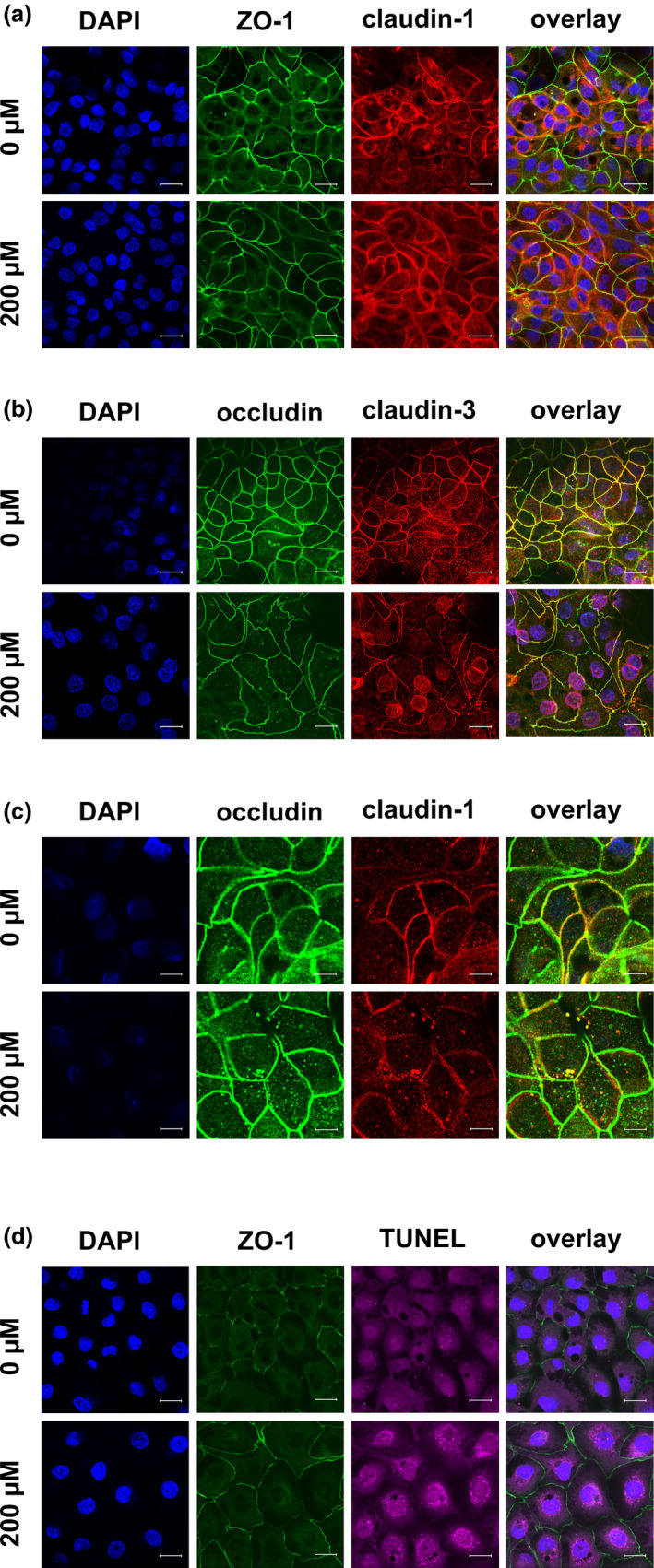
Immunocytological staining of IPEC‐J2 cells on filter membranes with antibodies raised against (a) ZO‐1 (green) and claudin‐1 (red), (b) occludin (green) and claudin‐3 (red), (c) occludin (green) and claudin‐1 (red), and (d) ZO‐1 (green) and TUNEL staining (purple) after 6 h of incubation with berberine. Nuclei were stained with DAPI (blue). (a) The signal for claudin‐1 was more intracellular than in the control. (b) The claudin‐3 signal became weaker in the lateral membrane and stronger inside the cell, especially around the nucleus. (c) Intracellular spots with co‐localized claudin‐1 and occludin were visible. (d) The cells were TUNEL positive (scale bar: (a, b & d) 20 µM, (c): 40 µM, *n* = 3, representative images)

### 3.6 Analysis of TJ proteins after 6 h

To investigate the dynamics of the changes in TJ protein quantity, localization, and apoptosis, a set of experiments was stopped after 6 h for protein isolation and immunocytological and TUNEL staining. No changes in the protein amount of claudin‐1, claudin‐3, occludin, and ZO‐1 could be observed after 6 h (data not shown). Immunocytochemistry revealed preliminary changes in the localization of claudin‐3, and occludin (Figure 9). Claudin‐3 signals became weaker in the lateral membrane and stronger inside the cell. Intracellular co‐localization of claudin‐1 and occludin can be seen in Figure 9c. The cells were TUNEL‐positive after 6 h of treatment with 200 µM berberine (Figure 9d).

## 4 DISCUSSION

In contrast to the previously reported barrier‐strengthening effect of berberine in Caco‐2 and HT29/B6 cells (Amasheh et al., 2010; Gu et al., 2009), we observed a decrease of the TEER in IPEC‐J2 cells after 6 h of incubation with 200 µM of berberine. After 24 h, all tested concentrations led to a dose‐dependent decrease of the TEER values compared with those of the controls. Moreover, we observed an increased paracellular flux of [^3^H]‐D‐Mannitol from the apical to basolateral compartments attributable to incubation with 200 µM berberine for 4 and 6 h. Because the reduction of barrier function was associated with 200 μM berberine incubation and rapidly intensified between 4 and 6 h, we carried out further analyses regarding the effects of berberine on apoptosis in IPEC‐J2 cells. An ApoToxGlo^TM^ Triplex assay was performed after 6 h of incubation with berberine concentrations between 25 and 200 µM, and the cell viability, toxicity, and induction of apoptosis were examined. The assay showed a dose‐dependent decrease of cell viability and an increase of caspase‐3 and −7 activity, representing the induction of apoptosis, while the toxicity was unchanged. The induction of apoptosis by berberine has been described in various tumor cell lines, for example, human oral epidermal carcinoma KB cells (Kuo et al., 2005), MG‐63 human osteosarcoma cells (Zhu et al., 2014), human breast cancer MCF‐7 cells (Pan et al., 2017; Sakaguchi et al., 2020), and human prostate carcinoma cell lines LNCaP, DU145, and PC‐3 (Mantena et al., 2006). The murine fibroblast cell line Balb/c 3T3 also exhibits induction of apoptosis by berberine (Yang et al., 1996).

Nevertheless, IPEC‐J2 is not a tumor cell line and is non‐infiltrative when grown in agar (Nossol et al., 2015) which is typical for tumor cells. Hence, the reason for the induction of apoptosis is unclear. Recently, (Zhu et al., 2020) reported inverse effects of berberine on cell viability in IPEC‐J2 cells. Concentrations between 25 and 250 µg/ml (corresponding to 67–670 µM) resulted in a dose‐dependent increase in cell viability as determined by MTT assay. Liu et al. (2019) used concentrations of berberine between 10 and 200 µg/ml (corresponding to 26.8–536 µM) and analyzed cell viability with Cell‐counting‐kit 8 (Dojindo, Japan): concentrations between 10 µg/ml and 100 µg/ml had no significant influence on cell viability, but 200 µg/ml led to a decrease. In contrast to our experiments, the cells described in both publications were cultured in fetal bovine serum (FBS), instead of porcine serum, which might be the reason for the different results. As we know from the work of Zakrzewski et al. (2013) reported in 2016, IPEC‐J2 cells cultured with porcine serum have more properties of intestinal epithelial cells than cells cultured with FBS.

Whereas the drop of the TEER to nearly zero after 24 h with 200 µM berberine might be regarded to occur because of the loss of cells and the formation of holes in the monolayer, the decrease after 6 h could be explained by the internalization of TJ proteins. This was supported by the increased [^3^H]‐D‐Mannitol flux after a 6‐h incubation with 200 µM berberine. Following a 24‐h incubation with berberine, we observed not only an internalization, but also a decrease in the total protein amount of typical intestinal barrier‐forming claudin‐1, claudin‐3, and occludin, and ZO‐1 was not significantly changed. In contrast, pore‐forming claudin‐2 was not detectable in IPEC‐J2, and claudin‐5 and −7 were not addressed but might be of interest for further studies.

However, the effects on the localization and amount of TJ proteins are in accordance both with a reduced barrier function and therefore to a reduced TEER and increased paracellular flux. The higher dose of berberine induced a stronger effect and also included cell loss. The 24‐h 50 µM berberine effect reflects a later endpoint of apoptosis, which was only reached slowly because of the lower concentration.

The consistent results with a stronger induction of apoptosis represent a proof‐of‐concept. Thus, quantification can be performed reliably by immunoblotting with densitometry in relation to total protein, as described in detail above.

In addition to the ApoToxGlo^TM^ Triplex assay, we used a TUNEL staining kit to visualize apoptotic cells. After 6 h of incubation, few TUNEL‐positive cells could be detected in cultures treated with 50 and 100 µM berberine, and with 200 µM berberine, most cells were TUNEL‐positive. After 24 h, TUNEL‐positive cells could be seen at all concentrations.

We suggest that IPEC‐J2 cells, cultured in same‐species serum, are more sensitive to berberine, and that the high doses that we used in our experiments might have been too high and might have over‐stimulated the cells leading to the induction of apoptosis. Moreover, a possible explanation for inducing apoptosis could be an internalization of claudin‐1 and occludin. The ability of occludin to induce apoptosis when occludin‐occludin or claudin‐claudin interactions are disrupted has been shown by Beeman et al. (2009, 2012). They induced the disruption of occludin, which led to an induction of apoptosis by the activation of the extrinsic pathway, including the activation of caspase‐3. Furthermore, they analyzed the connection between claudin‐4 disruption and apoptosis induction; as occludin plays an important role in the induction of apoptosis when claudins are disrupted. In addition, a non‐junctional co‐localization of occludin and claudin‐4 inside the cells was also observed. Further analysis revealed that the proteins are also co‐localized with the death‐inducing signaling complex (DISC). This is in accordance with the findings in our experiments, as the images show a similarity regarding the co‐localization of occludin and claudin‐1. Moreover, caspase‐3 was also found inside the DISC‐claudin‐occludin‐complex by Beeman et al. (2009, 2012) and the ApoToxGlo^TM^ Triplex assay that we performed revealed the activation of caspase‐3 (and −7) by berberine. Hence, we suggest that higher berberine concentrations in IPEC‐J2 cells cultivated in porcine serum induce the disruption of claudin‐claudin interactions, leading to the induction of apoptosis. Additional detailed mechanistic investigations regarding the way that berberine causes a downregulation of junctional protein expression and internalization should be subject of further studies.

The effects on cell viability are in accordance with the observation that secondary plant compounds induce adverse reactions at higher concentrations; these effects limit therapeutical use and therefore are of significant physiological and pathological relevance. Although the susceptibility of IPEC‐J2 cells to various agents can be compared with that of human models (Gunzel & Yu, 2013; Schierack et al., 2006), a higher sensitivity of IPEC J2 cells for berberine compared with cancerogenic epithelial cell models such as Caco‐2 or HT29/B6 cannot be completely ruled out. Therefore, in future approaches, experiments should be extended to even lower concentrations of berberine. For the time being, however, a beneficial effect cannot be concluded for porcine jejunal cells. Further research is needed to clarify and establish the signaling of apoptosis induction by berberine in IPEC‐J2 cells and intestinal tissue in more detail.

## CONFLICTS OF INTEREST

The authors declare that the research was conducted in the absence of any commercial or financial relationships that could be construed as a potential conflict of interest.

## AUTHORS’ CONTRIBUTIONS

The research project was planned by V. Cornelius and S. Amasheh. Experiments were carried out by V. Cornelius and L. Droessler. Data analysis was performed by V. Cornelius. The article was written by V. Cornelius, L. Droessler and S. Amasheh.

## Supporting information



Fig S1

Table S1‐S4

## Data Availability

Data available upon request.
