# Supplementary figures and images for: Concerted action of berberine in the porcine intestinal epithelial model IPEC‐J2: Effects on tight junctions and apoptosis
Source: Physiol Rep. 2022 Apr 5;10(7):e15237. doi: 10.14814/phy2.15237 (PMC8981188; doi:10.14814/phy2.15237)

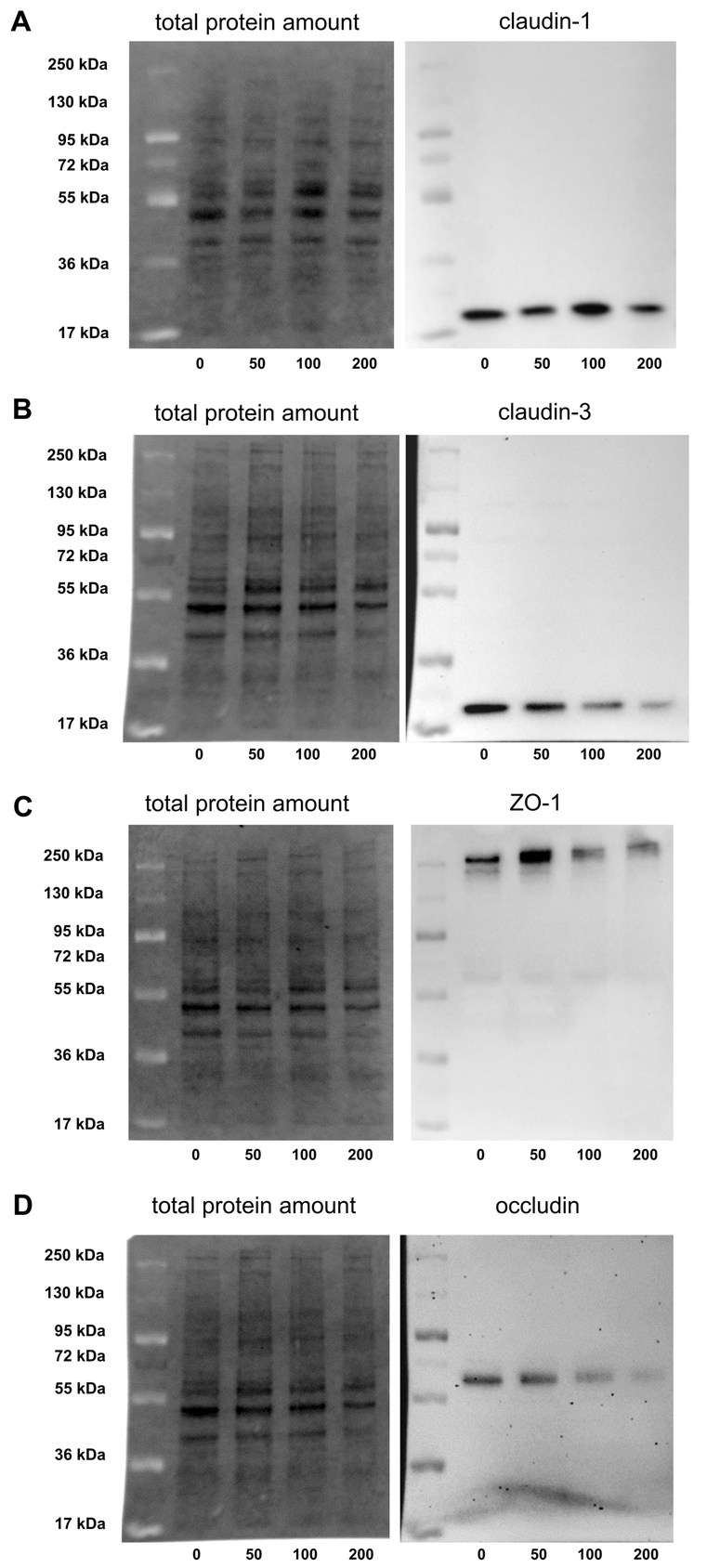

Supplement: Supplementary file 1 — Fig S1 [file PHY2-10-e15237-s001.jpg]
