# Supplementary material for: Concerted action of berberine in the porcine intestinal epithelial model IPEC‐J2: Effects on tight junctions and apoptosis
Source: Physiol Rep. 2022 Apr 5;10(7):e15237. doi: 10.14814/phy2.15237 (PMC8981188; doi:10.14814/phy2.15237)
Supplement: Supplementary file 2 — Table S1‐S4 [file PHY2-10-e15237-s002.docx]

**Suppl. Table 1** **Effects of berberine on IPEC-J2 cells.** Berberine was used at concentrations of 50, 100 and 200 µM. Values are means ± SEM. Significance is given versus control (Dunnett´s test for multiple comparisons). ns, not significant; * = *p* < 0.05; *** = *p* < 0.001.

| One-way ANOVA | **4 h**  F (3,66) = 8.88,  *p* < 0.0001, n = 16-19 | **6 h**  F (3,56) = 21.48,  *p* < 0.0001, n = 15 | **24 h**  F (3,54) = 116.64,  *p* < 0.0001, n = 13-16 |
| --- | --- | --- | --- |
| Berberine concentration (µM) | % of initial resistance | | |
| 0 | 95.6 ± 6.3 | 92.5 ± 4.1 | 120.4 ± 6.6 |
| 50 | 112.9 ± 5.8 ns | 108.6 ± 5.0 ns | 82.4 ± 7.4*** |
| 100 | 98.4 ± 7.5 ns | 80.8 ± 8.7 ns | 11.9 ± 3.1*** |
| 200 | 71.7 ± 4.3* | 42.0 ± 5.6*** | 0.8 ± 0.3*** |

**Suppl. Table 2 Paracellular permeability measurements.**Berberine was used at concentrations of 50, 100 and 200 µM. Values are medians and interquartile range (IQR). Significance is given versus control (Dunn´s test for multiple comparisons). ns, not significant; *** = *p* < 0.001.

| Kruskal-Wallis | **2 h**  Chi²= 3.01, *p* = 0.39,  df = 3, n = 5-6 | **4 h**  Chi²= 12.9, *p* = 0.0049,  df = 3, n = 5-6 | **6 h**  Chi²= 18.5, *p* = 0.0003,  df = 3, n = 5-6 |
| --- | --- | --- | --- |
| Berberine concentration (µM) | [^3^H]-D-Mannitol flux rate (nmol/cm²/h) | | |
| 0 | 0.08 (-0.03 - 0.10) | 0.1 (0.08 – 0.15) | 0.09 (0.04 – 0.25) ns |
| 50 | 0.06 (0.02 – 0.11) ns | 0.12 (0.09 – 0.16) ns | 0.21 (0.19 – 0.24) ns |
| 100 | 0.12 (0.02 – 0.77) ns | 0.63 (0.26 – 1.9) ns | 2.72 (1.98 – 3.31) ns |
| 200 | 0.11 (0.06 – 0.23) ns | 1.54 (0.73 – 2.21) ** | 9.54 (6.6 – 13.56) *** |

**Suppl. Table 3 Results of the ApoToxGlo^TM^ Triplex assay*.***Berberine was used at concentrations of 25, 50, 75, 100, 150 and 200 µM. Values are means ± SEM. Significance is given versus control (Dunnett´s test for multiple comparisons). ns, not significant; * = *p* < 0.05; ** = *p* < 0.01; *** = *p* < 0.001.

|  | **Viability** | **Cytotoxicity** | **Caspase 3/7 activity** |
| --- | --- | --- | --- |
| One-way ANOVA | F (7,24) = 4.42,  *p* = 0.0028, n = 4 | F (7,24) = 0.37,  *p* = 0.9061, n = 4 | F (7,23) = 14.48,  *p* < 0.0001, n = 3 - 4 |
| Berberine concentration (µM) | % of control (medium) | | |
| 0 | 94.5 ± 7.4 ns | 99.3 ± 9.8 ns | 153.5 ± 27.9 ns |
| 25 | 83.5 ± 7.0 ns | 106.0 ± 13.0 ns | 225.8 ± 44.7* |
| 50 | 79.2 ± 9.7 ns | 107.9 ± 17.9 ns | 286.6 ± 24.6*** |
| 75 | 71.1 ± 11.8 ns | 124.6 ± 27.7 ns | 326.1 ± 26.1*** |
| 100 | 67.5 ± 8.7* | 101.5 ± 23.6 ns | 318.1 ± 12.9*** |
| 150 | 61.3 ± 2.5** | 93.7 ± 13.8 ns | 373.7 ± 30.0*** |
| 200 | 57.3 ± 2.5** | 90.0 ± 16.1 ns | 405.6 ± 31.2*** |

**Suppl. Table 4 Densitometry of tight junction proteins.**Berberine was used at concentrations of 50, 100 and 200 µM. Values are means ± SEM. Significance is given versus control (Dunnett´s test for multiple comparisons). ns, not significant; * = *p* < 0.05; ** = *p* < 0.01; *** = *p* < 0.001.

| One-way  ANOVA | **claudin-1**  F (3,19) = 6.51,  *p* = 0.003, n = 5-6 | **claudin-3**  F (3,16) = 30.4,  *p* < 0.0001, n = 5 | **ZO-1**  F (3,18) = 2.7,  *p* = 0.08, n = 5-6 | **occludin**  F (3,12) = 18.5,  *p* < 0.0001, n = 4 |
| --- | --- | --- | --- | --- |
| Berberine concentration (µM) | % of control | | | |
| 50 | 45.0 ± 8.5** | 37.4 ± 8.2*** | 145.6 ± 34.8 ns | 49.1 ± 14.1** |
| 100 | 82.4 ± 21.9 ns | 27.0 ± 9.5*** | 59.9 ± 14.7 ns | 26.8 ± 6.4*** |
| 200 | 41.3 ± 9.0** | 14.2 ± 5.9*** | 61.4 ± 20.4 ns | 24.2 ± 5.2*** |
